# An Experimental Study on the Mechanical and Biological Properties of Bio-Printed Alginate/Halloysite Nanotube/Methylcellulose/Russian Olive-Based Scaffolds

**DOI:** 10.15171/apb.2018.073

**Published:** 2018-11-29

**Authors:** Babak Roushangar Zineh, Mohammad Reza shabgard, Leila Roshangar

**Affiliations:** ^1^Mechanical Engineering Department, University of Tabriz, Tabriz, Iran.; ^2^Stem Cell Research Center, Tabriz University of Medical Sciences, Tabriz Iran.

**Keywords:** Halloysite Nanotube, Alginate, Methylcellulose, Scaffold, Bio print, WST assay

## Abstract

***Purpose:*** Cartilage shows neither repairs nor regenerative properties after trauma or gradual wear and causes severe pain due to bones rubbing. Bioprinting of tissue-engineered artificial cartilage is one of the most fast-growing sciences in this area that can help millions of people against this disease.

***Methods:*** Bioprinting of proper bioscaffolds for cartilage repair was the main goal of this study. The bioprinting process was achieved by a novel composition consisting of alginate (AL), Halloysite nanotube (HNT), and methylcellulose (MC) prepared in bio-ink. Also, the effect of Russian olive (RO) in chondrocytes growth on bioscaffolds was also investigated in this work. Compressive, hardness and viscosity tests, Energy-Dispersive X-Ray Spectroscopy (EDX), Fourier-Transform Infrared Spectroscopy (FT-IR), Differential Scanning Calorimetry (DSC), water-soluble Tetrazolium (WST) assay, and also transmission electron microscopy (TEM) and scanning electron microscopy (SEM) were carried out.

***Results:*** The results show that in constant concentrations of AL, MC, and RO (20 mg/ml AL, 20 mg/ml MC, and 10 mg/ml RO) when concentration of HNT increased from 10 mg/ml (T-7) to 20 mg/ml (T-8) compressive stiffness increased from 241±45 kPa to 500.66±19.50 kPa. Also, 20 mg/ml of AL in composition saved proper water content for chondrocyte growth and produced good viscosity properties for a higher printing resolution.

***Conclusion:*** RO increased chondrocytes living cell efficiency by 11% on bioprinted scaffolds in comparison with the control group without RO. Results obtained through in-vivo studies were similar to those of in-vitro studies. According to the results, T-7 bio-ink has good potential in bioprinting of scaffolds in cartilage repairs.

## Introduction

The goal of tissue engineering is to produce bioscaffolds with good tolerance against existing mechanical forces and biocompatible properties such as living tissues without any toxicity.^1-3^ Bioprinting process is one of the best methods to produce bioscaffolds with living cells. High accuracy in printing dimensions, homogenous dispersion of cells through the scaffold, and excellent control during the printing process make the bioprinting favorable to tissue engineering scientists.^4^ The bioprinting idea is based on the printing of biomaterials and living cells together. Today, the challenges in bioprinting are producing proper biomaterial that can increase the cell living efficiency besides its good mechanical properties similar to living tissues and suitable properties for high-resolution printing such as proper viscosity.^5^ Alginate (AL) as a compound with good properties during the bioprinting process such as satisfactory mechanical properties and proper compressive modulus can be used in knee cartilage repairs.^6^ AL changes to a stable hydrogel through ionic interaction between carboxyl groups of alginate and Ca^2+^.^7,8^ Biocompatibility of AL and its controllable viscosity improve its applications in scaffolding and bioprinting.^9^ Halloysite is a biocompatible nanoclay.^10^ The main advantage of halloysite is that it can be used easily after washing and sterilization in bioprinting and cell culture structures, making it much cheaper than other nanofillers.^11^ Addition of 4-5 wt.% halloysite nanoclay can increase material mechanical strength between 50-80%, suggesting its perfect potential for an appropriate additive in bioscaffolds and bioprinting.^10^ Nanofibers of cellulose are beneficial in scaffolding because of their biocompatibility and good mechanical characteristics. Methylcellulose (Mc) as a favorable material for cell culture and scaffolding could increase bioprinting efficiency.^12^ Russian olive (Elaeagnus angustifolia L.) fruit and seed powder are traditionally used for the healing of knee and cartilage defects.^13,14^ Russian olive (RO) fruit contains sugar, vitamins (such as tocopherol, vitamin C, B1), and minerals such as (calcium, magnesium, potassium, and iron). These vitamins and minerals are very beneficial for chondrocytes growth and their proliferation.^15^ Ming Xian Liu et al.^16^ studied several compositions consisting of alginate/halloysite nanotube (HNTs) composite bioscaffolds produced by the freeze-drying method. HNTs is mixed with alginate for enhancing both the mechanical and biological properties of freeze-dried bioscaffolds. HNTs that are partly oriented in a zigzag form in the composite scaffolds increase the density and water-swelling ratio of the composition. The results revealed that fibroblast cells of the mice attach effectively to the alginate/HNT scaffolds surface and the pure alginate and shows potential for tissue engineering applications. Afshar et al.^17^ investigated Aminated chitosan/alginate scaffold containing halloysite nanotubes composition for improving cell attachments to the freeze-dried scaffolds. The results show that the scaffolds compared of chitosan/alginate/halloysite have a high cell proliferation and cell attachments with L929 cells to the scaffolds. Huijun Li et al.^18^ studied alginate/methylcellulose composition to increase the adhesion between three-dimensional bioprinted layers of bioscaffolds and revealed that the novel Alg/MC composition has a potential to be used in bioprinting of tissues. Biao Huang et al.^19^ studied sodium alginate (SA)/halloysite nanotubes (HNTs) composites and reported that HNTs can improve the mechanical properties of composite hydrogels. Their results also revealed that the SA/HNTs biomaterial with 80% HNTs has a compressive stress of 2.99 MPa, while the compressive stress of pure sodium alginate hydrogel is 0.8 MPa at 80% strain. Anuj Kumar et al.^20^ studied the effects of CNCs and/or HNTs on alginate based bioscaffolds and reported the improved mechanical, thermal, and cytocompatibility properties of scaffolds. Yildirim et al.^21^ studied alginate and single-walled carbon nanotube (SWCNT) scaffolds produced by a freeform fabrication technique. They reported some mechanical improvements in the alginate when mixed with SWCNT and suggested the great potential of alginate/SWCNT bioscaffolds for bioscaffolding.

According to previous studies, bioprinting with AL/HNT/MC has not yet been performed. We added RO fruit and seed powder to bio-ink for studying its effect on cells living performance. AL can increase the water content and formability of biomaterial. Addition of MC not only increases mechanical properties of biomaterial but also produce micropores during cell cultivation period. HNT also increases the mechanical endurance of biomaterial. Finally, RO due to its specific composition could increase the biological performance of bioscaffolds. Different mechanical and biological tests such as compressive, hardness and viscosity tests, scanning electron microscopy (SEM), inverted microscopy, Energy-Dispersive X-Ray Spectroscopy (EDS), Fourier-Transform Infrared Spectroscopy (FT-IR), Differential Scanning Calorimetry (DSC), vital staining of cells via cell tracer, and WST assay were carried out to characterize the properties of the developed bio-ink.

## Materials and Methods

Alginic acid sodium salt with a molecular weight of 150-250 kda, methylcellulose with a molecular weight of 90 kda, phosphate buffered saline (PBS-containing sodium chloride (8 g/L), sodium phosphate (0.24 g/L), potassium chloride (0.2 g/L) and potassium phosphate (1.42 g/L)), trypsin, and WST were purchased from Sigma Aldrich (Munich, Germany). Halloysite nanotube with a molecular weight of 294 kda was purchased from natural-nano (Rochester, NY, USA). Lipophilic vital dye Dil (Biottium, INC, Hayward, USA) and Human chondrocytes were purchased from Pasteur Institute Tehran (Iran). Dried RO powder was purchased from Biology Institute of Tabriz Medical University (Iran). Fetal bovine serum was purchased from Life Technologies (Grand Island, NY, USA) and phosphate buffer was purchased from Proscitech, Thuringowa (Australia). Araldite, uranyl acetate, lead citrate, and aqueous osmium tetroxide were purchased from TAAB, Berkshire (UK). HEPES buffered saline (HBS-10 g/L HEPES, 16 g/L NaCl, 0.74 g/L KCl, 0.27 g/L Na_2_HPO_4_.2H_2_O and 2.0 g/L dextrose), Paraformaldehyde (PFA), uranyl acetate and Araldite purchased from Sigma Aldrich (Munich, Germany). Glutaraldehyde was purchased from Proscitech (Munich, Germany). Dulbecco’s Modified Eagle’s Medium (DMEM), Ham’s F12, fetal bovine serum, L-ascorbate, penicillin, streptomycin, and fungizone were purchased from Gibco (UK). Lipophilic vital dye Dil was purchased from Biottium (INC, Hayward, USA). Finally, toluidine blue, acetone, BSA plus, and 2-(4-Amidinophenyl)-6-indole carb amidine dihydrochloride (DAPI) were purchased from Sigma Aldrich (Munich, Germany).

### Preparation of biomaterial

Alginate and MC solutions were prepared in the concentrations of 10 mg/ml and 20 mg/ml for AL and 10 mg/ml and 20 mg/ml for MC in phosphate buffered saline (PBS). Then, different percentages of AL and MC solutions were mixed in a ratio of 1:1 and stirred for 4 h. Afterward, 10 mg/ml and 20 mg/ml of HNT powder were prepared in deionized water and added to these solutions and incubated under stirring overnight. The prepared bio-inks (with different compositions) were added to the solutions of fruit and seed powder of RO with a concentration of 10 mg/ml. All experiments were performed at room temperature (37 °C). Table 1 shows prepared bio-inks with different compositions.

**Table 1:** Prepared bio-inks with different compositions

BIO-INK	Alginate (mg/ml)	Methylcellulose (mg/ml)	Halloysite (mg/ml)	RO (mg/ml)
T-1	10	10	10	10
T-2	10	10	20	10
T-3	10	20	10	10
T-4	10	20	20	10
T-5	20	10	10	10
T-6	20	10	20	10
T-7	20	20	10	10
T-8	20	20	20	10

### Bioprinting of cartilage scaffolds and experimental tests

**Figure 1:**
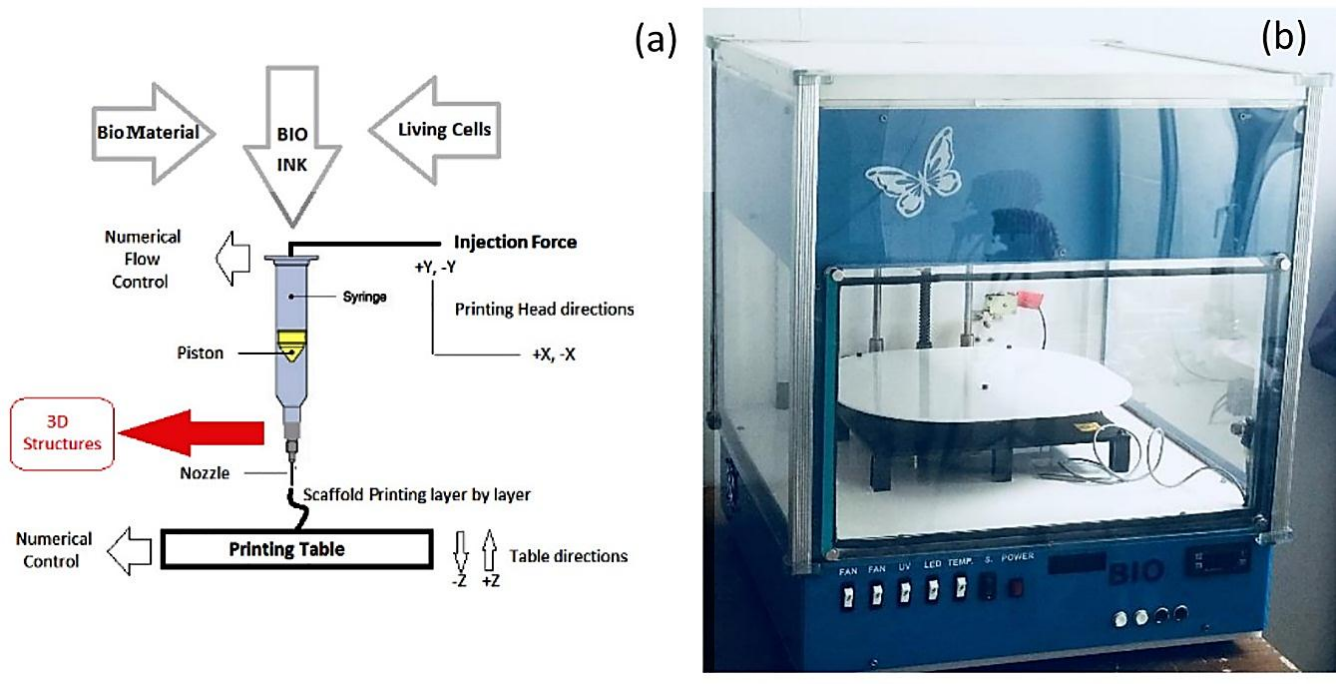
Bioprinter used during the tests (a) A schematic of bioprinter (b) The bioprinter used for experimental tests

Injection based bioprinter manufactured in our lab and used for scaffold bioprinting. The bioprinter shown in Figure 1 has a high accuracy, trustable sterile cartridge, and filtering fans for clean air circulation. Chondrocytes were dissociated into single cells and gently mixed with prepared bio-inks. The final concentration was 5 ×10^6^ cells/ml. The mixture was sterilized using ultraviolet radiation. Layers were Perpendicular to each other. The thickness of printing layers was 0.5 mm without line spacing. Bioprinting was done using a 0.5 mm needle. Bioprinting speed of 10 mm/s and feed rate of 5mm^3^/s were selected. Scaffolds were printed with 5 layers, 20 rows, and 20 columns. Bioprinted scaffolds were then transferred into glass plates and placed in an incubator. Next, a 100 mM CaCl_2_ solution was added over scaffolds for 8 min and ionic interaction occurred between the alginate chains and Ca^2+^.

#### Viscosity investigation

The solutions with different composition of bio-inks and cells were used for measurement of viscosity. The kinematic viscosity of different solutions was measured using ATAGO viscometer BASE plus (Japan) at room temperature with three repetitions. The viscosity of the bio-inks without living cells was identified in the same condition.

#### Scanning electron microscopy

Scaffolds were subjected to SEM using a Mira3 TESCAN (Czech). Before the SEM analysis, the specimens were washed twice with HEPES buffered saline (HBS) and dried samples were coated with gold for 300 seconds with sputtering coater and prepared for imaging.

#### Energy-Dispersive X-Ray Spectroscopy

In order to prepare biomaterials for EDS (Mira3 TESCAN), dried samples were placed in the EDX equipment to determine the elemental composition of biomaterials.

#### Transmission electron microscopic analysis

For ultrastructural studies, cultured chondrocytes were fixed in 2.5% glutaraldehyde in a 0.1M phosphate buffer and post-fixed in 1% aqueous osmium tetroxide. The samples were then dehydrated in the graded concentration of ethanol and embedded in Araldite. Ultra-thin sections from selected blocks were treated with uranyl acetate and lead citrate and observed in (LEO 906) TEM (Oberkochen, Germany).

#### Differential Scanning Calorimetry

The thermal characterization of composition was obtained with a differential scanning calorimeter (DSC), Linseis PT100 (Germany). The samples heating rate was 10 °C/min–1 from 0 °C to 120 °C. The N_2_ atmosphere with a flow rate of 20 ml/min was used for tests.

#### Compressive characterization

Compressive tests were performed by GOTECH AI-7000M (Taiwan) on freeze-dried solid scaffolds at a 2.00 mm/min loading speed by averaging of three repetitions (n=3). Samples were cut into 40 mm diameter and 20 mm height. The unconfined compressive tests were performed according to ASTM D695 at 90% strain at 37 °C.

#### Hardness determination

The solid model was used to determine the hardness of material at 37 °C. In each sample shore, a hardness was calculated according to ASTM D2240 with five repetitions.

#### Fourier transform infrared

Bruker (Germany) spectrometer was used to take spectra of T-7 bio-ink. Next, 2 mg sample of 200 mg kbr pallets were prepared and subjected to scanning within a range of 400- 4000 cm-1.

#### Thermogravimetric analysis (TGA)

Thermal stability of the bio-ink gel studied in this test. Gel bio-ink was freeze at -60 °C and then dried in vacuum for 96 h. the grounded gel (8 mg in weight) was tested in platinum plate. TGA test was carried out with Linseis (L81A1750-USA) at a heating rate of 8 °C/minin nitrogen atmosphere from 20 °C to 800 °C.

#### Cell culture

Chondrocytes were cultured in a special media containing Dulbecco’s Modified Eagle’s Medium (DMEM) and Ham’s F12 (1:1 mixture) supplemented with 10% fetal bovine serum, 50 μg/μl L-ascorbate, 100 μg/μl penicillin, 100 μg/ml streptomycin, and 2.5 μg/μl fungizone, and then incubated in a humidified incubator at 37 °C with 5% CO_2_. Chondrocytes were expanded to 80% confluency and then dissociated. About 5 × 10^6^ chondrocytes per ml of bio-ink were used in this study.

#### Cell cytotoxicity assay

Cytotoxicity assay was conducted using the WST assay. The samples were incubated for 24, 48, 72, and 96 h at 37 °C and 5% CO_2_. For WST assay, the medium of the printed construct in each group was replaced with a WST for 1h. The absorbance at 480 nm wavelength was detected using an ELISA plate reader (BioTek, VT, USA) at 600 nm reference wavelength. Cells with blue stain represented viable cells and revealed their distribution in the bio-ink. Samples were transferred into PBS for light microscopy analysis (Olympus, Japan).

#### Cell viability study

CM-Dil staining method^22^ was used to monitor the live cells. The 3D cell-tracer construct was cultured for 1, 6, and 20 days in a chondrocyte medium labeled with 2µm cell tracer TM CM-Dil. Suspended chondrocytes at a density of 1 × 10^6^ cell/ml were labeled with 5 μl/ml of Dil construct agent. Cells were incubated with a labeled solution at 37 °C for 20 min and studied with a fluorescent microscopy apparatus (Olympus, Japan).

#### Histological analysis

Cultured chondrocytes were fixed in 4% paraformaldehyde and kept frozen until sectioning. Then they were sectioned using cryostat and stained with toluidine-blue and safranin 0-fast green, according to previous studies.^23^ Histological analyses were carried out using light microscopy.

#### Transplantation of chondrocyte-seeded construct

To transport chondrocyte-seeded construct, 10 male sheep (Bergamasca–Massese) were purchased. Sheep were sedated with Xylazine hydrochloride (HCl) (0.1 mg/kg sheep) for a surgical procedure. Both knee joints were exposed via a lateral Para patellar skin incision. Two full-thickness cartilage defects (4 mm in diameter and 1 mm in depth)^24^ were created in each joint. The chondrocyte-seeded construct was fashioned in the defect as much as possible. The defect in both knees was filled with hydrogel and sealed with fibrin glue (Tisseel™Baxter AG) for 6 months. Right knee was chosen as the control group and left knee was chosen as the treatment group. The sheep were maintained for 6 a month post-treatment and then the specimens were transferred to the laboratory.

#### Toluidine blue staining

In this step, 1% of toluidine blue was used for staining and extra dye were washed by PBS and then mounted with Canada balsam.

#### Immunofluorescence staining of collagen Type 2

We cut 10 μm thick sections by cryostat, fixed them using -20 °C acetone, and washed three times with PBS (10 mm sodium phosphate, 0.15 M sodium chloride, and pH 7.4). The slides were incubated with a solution of 1% BSA plus 10% goat serum for 2 h. Then, the primary antibody was added and incubated for 18 h at 4 °C. The secondary antibody was added for 1 h and then 4′,6-diamidino-2-phenylindole (DAPI) was added to each section. The slides were protected from light and incubated for 10 min at room temperature and were washed twice, 5 min each, in PBS. Morphological analyses were conducted with fluorescent microscopy.

#### Statistical analysis

The Mean ± standard deviation (SD) was used for data expression. Excel statistical software and GraphPad Prism 7 were used for data analysis. An independent sample t-test was used for viscosity studies. All data are the average of three repetitions in each test. Repeated measurement analysis was used to determine statistical significance in compression and WST tests results. All analyses were performed at a significance level of p < 0.05.

## Results and Discussion

### Bioprinting of 3D scaffolds containing chondrocytes

**Figure 2:**
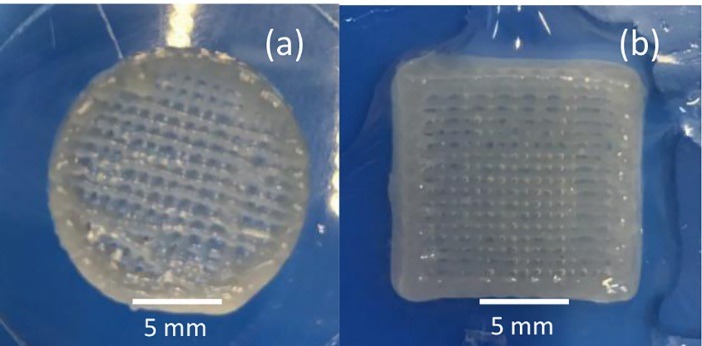
Printed scaffolds(a) A scaffold printed without living cells (b) A scaffold printed with living cells

The prepared bio-inks were mixed slowly with chondrocyte in concentration of 5 × 10^6^ /cm^2^. Chondrocytes were mixed for 150 seconds and then filled in sterilized cartridge for printing. Scaffolds with different composition were printed (Figure 2). Bio-ink containing 30 mg/ml alginate, 50 mg/ml HNT, and 30 mg/ml methylcellulose solutions show the best performance in bioprinting and saved the printing shape very well. A high percentage of alginate increased the gel form of bio-ink and improved printing resolution. Existence of RO powder (10 mg/ml) had no significant effect on printing results of scaffolds.

### Microstructure and composition

**Figure 3:**
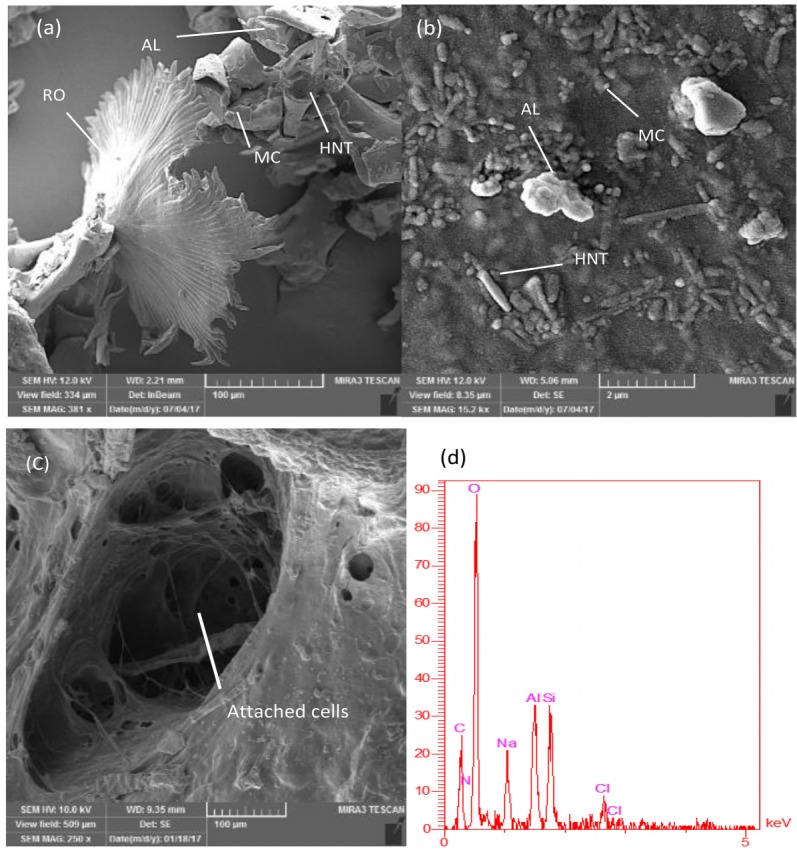
Scanning electron microscopy images of Methylcellulose particles, Halloysite nanotubes, and RO powder(a) RO, alginate, methylcellulose, and HNT at the surface (b) the zig-zag combination of HNT in bio-ink surface (c) cells attached at the surface of bioprinted scaffolds, and (d) Energy dispersive x-ray of T-7 biomaterial.

Using SEM images with high magnifications (Figure 3a) showed a homogenous composition, a high percentage of HNT, and RO particles at the surface of the dried samples. MC particles, Nanotubes of HNT in parallel and zigzag forms with each other and RO powder can be seen in Figure 3b. Also, dried samples of 24 h cell cultured scaffolds showed a strong attachment of cells to the surface of bioprinted scaffolds in SEM images (Figure 3c). Also, EDS results show Aluminum (Al), sodium (Na), Silicon (Si), and chlorine (Cl) in the composition sourced from a constituent of the biomaterial.

### Compressive investigation

**Figure 4:**
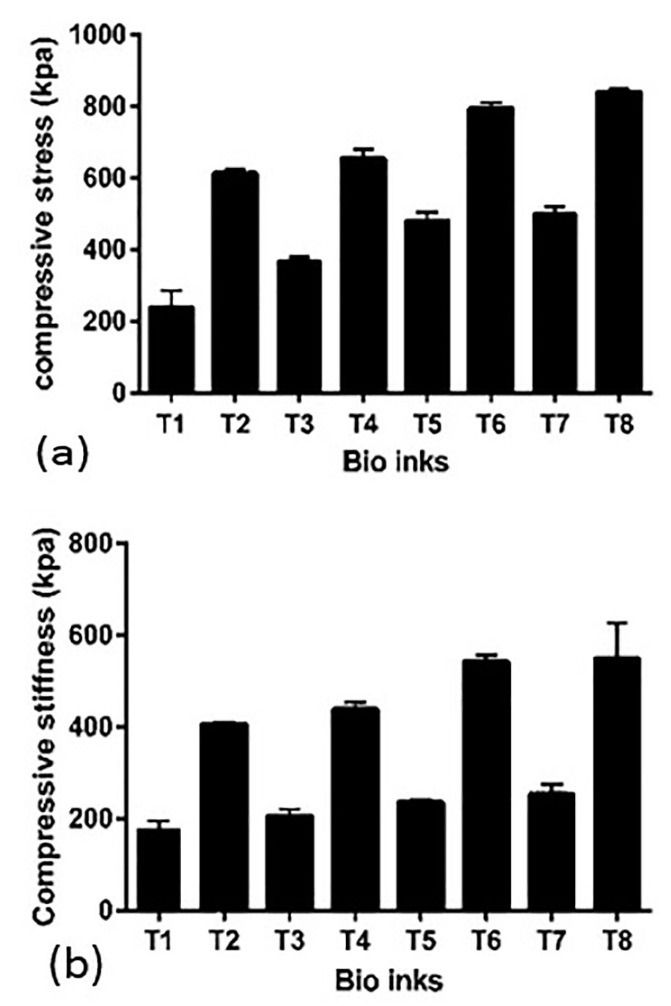
Compressive properties of bioprinted scaffolds (a): Compressive stress increased from T-1 to T-8 according to alginate and halloysite Nano tube content of biomaterials. (b): Compressive stiffness increases from T-1 to T-8 due to a different percentage of alginate and HNT in the structure.

Different amounts of HNT and AL alter compressive properties of bioprinted scaffolds. It is seen from Figure 4 that 20 mg/ml of HNT and 20 mg/ml of AL (T-8) compressive properties are higher in comparison with 10 mg/ml of HNT and 10 mg/ml of AL (T-1). Also, the T-7 group shows a higher compressive stress (210%) in comparison to the T-1 group. Compressive stiffness continually grows by increasing the amounts of HNT and AL; however, HNT has a higher effect on compressive stiffness than AL. Results revealed that existence of methylcellulose and RO powder did not change the compressive properties of different bio-inks significantly.

### Kinematic viscosity investigation

The kinematic viscosity of different bio-inks with different compositions is shown in Table 2. The kinematic viscosity decreases by adding cells to each group. Best results of bioprinting were achieved in viscosities between 2000-2400 mm^2^/s.

**Table 2:** Kinematic viscosity of bio-inks with and without living cells

Bio-ink	Kinematic Viscosity (mm2/s) Without cells	Kinematic Viscosity (mm2/s) With cells
T-1	1200	1100
T-2	1400	1300
T-3	1600	1500
T-4	1800	1700
T-5	1900	1800
T-6	2100	2100
T-7	2300	2200
T-8	2500	2300

### Hardness investigation

The hardness of different composition of solid materials was measured at different points of each sample (n=5) and the mean hardness for each one was calculated (Table 3). By increasing the amounts of alginate and HNT in the composition of bio-inks shore A, hardness increases continually.

**Table 3:** Different results of shore A hardness test from bio-ink samples (n=5)

Bio-ink	Shore A hardness
T-1	53.6± 0.894
T-2	57.8± 0.836
T-3	60± 0.707
T-4	60.8± 0.836
T-5	65.6± 0.547
T-6	68.4 ± 1.140
T-7	71±1.000
T-8	77± 1.581

### Differential Scanning Calorimetry (DSC)

**Figure 5:**
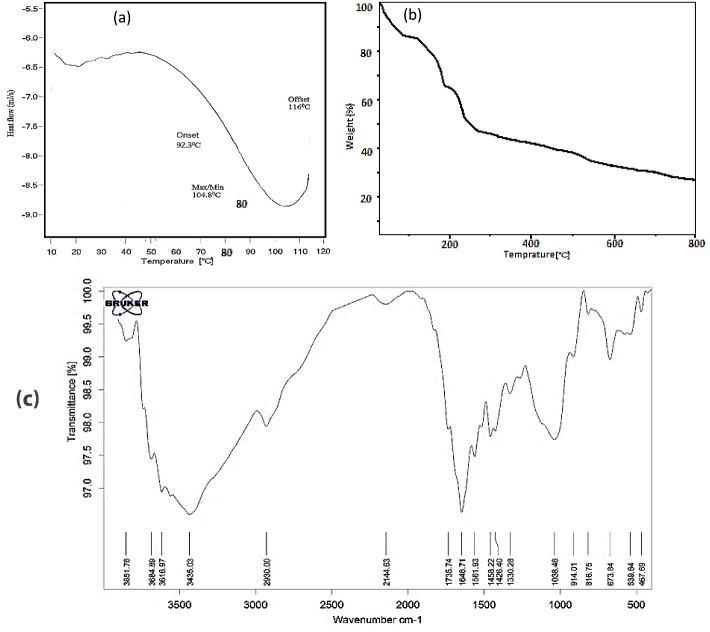
(a): Differential Scanning Calorimetry results of T-7 Bio-ink, (b): Thermogravimetric analysis curves of T-7 bio-ink (c): Fourier transform infrared results of T-7 Bio-ink.

The DSC tests were taken between 0 and 120 °C. This range was chosen because of working temperature of bioscaffolds. It is seen that there is no exothermic or endothermic peak in working range but an endothermic peak seen in 104.8 °C due to a water loss of the samples. Figure 5a presents the DSC analysis of T-7 biomaterial.

### Thermogravimetric analysis

Figure 5b shows the TGA curves of T-7 bio-ink in a nitrogen atmosphere from ambient temperature to 800 °C. The TGA curve of T-7 shows three mass losses and degradation steps. The first step was about 13% before 100 °C due to evaporation of physically bound water in alginate and bio-ink structure. The mass loss of about 35% around 190 °C for bio-ink was related to the decomposition of alginate macromolecular chains in bio-ink composition. eventually, The TGA curve of T-7 shows almost constant decreasing from 500 to 800 °C.

### Fourier transform infrared (FT-IR)

FT-IR results (Figure 5c) show that the peaks at 3400-3900 cm^−1^ (3435.03 cm^−1^ for T-7) are related to O-H stretching bonds of Alginate and Methylcellulose. Also, in the range from 2850 to 3000 cm^−1^ (2930.00 cm^−1^ for T-7), stretching vibration C–H can be seen. A peak at 2144.63 cm^−1^ is indexed to Si-H bonds related to the existence of HNTs in the structure. Peaks at 1646.71, 1735.74, and 1561.93 cm^−1^provide an evidence for existence of carboxylic acid salt (-COO asymmetric stretch, 1500-1650 cm^−1^).^25^ In addition, a peak at 1426.40 is related to C–C stretching; peak at 1458.22 is attributed to CH2 and CH3 bonds; and the peaks at 1038.46 indexed to C–O, 816.75 and 914.01 are indexed to C–C–H, and 673.84 cm^−1^ is indexed to C–O–H stretching vibration bonds.^26^

### Histological analysis

**Figure 6:**
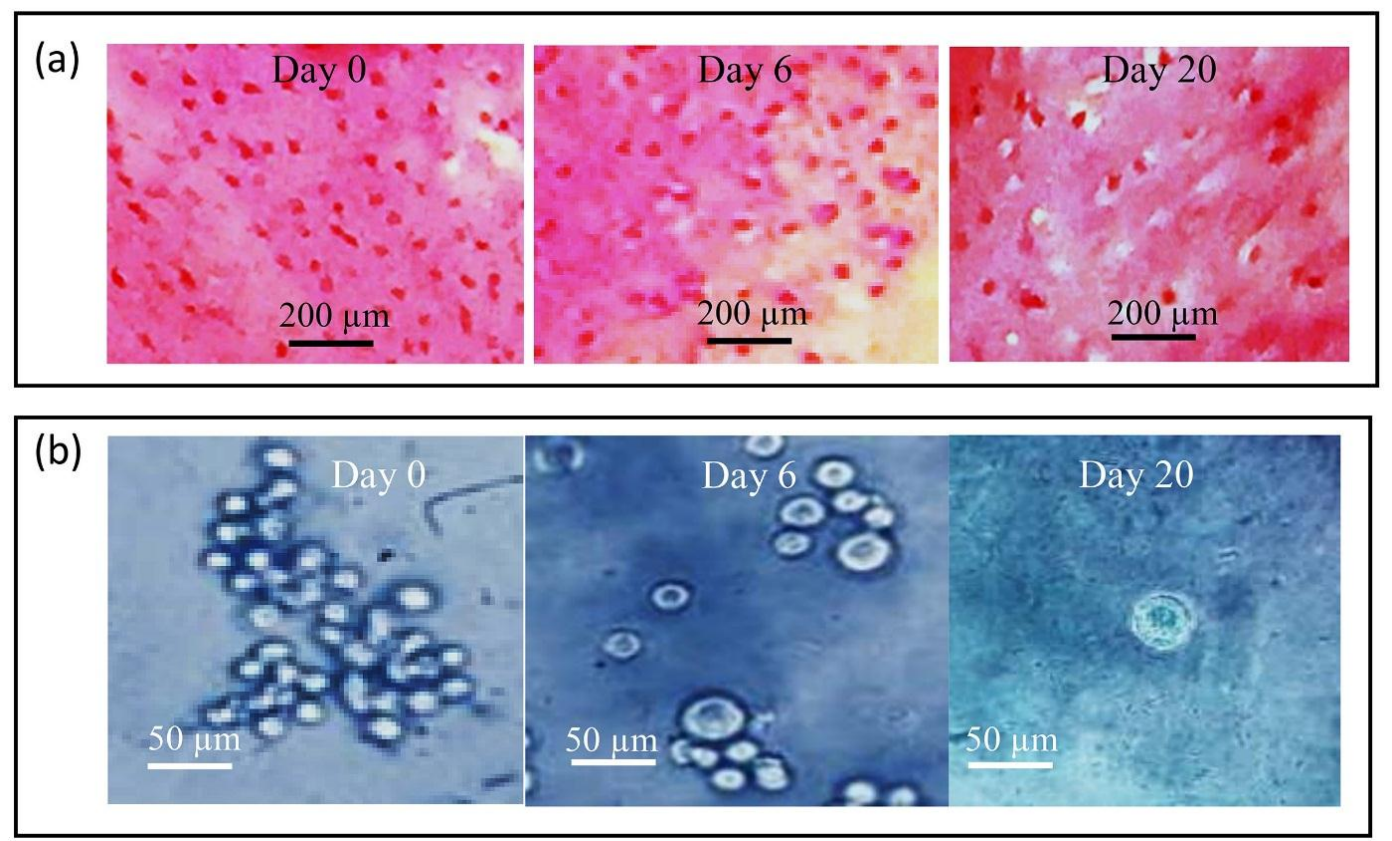
(a): Safranin-o staining of printed chondrocyte-seeded constructs in 0, 6, and 20 days’ culture from the T-7 group. Note the increase in cell size and highly positive stain for safranin-o in day 20 culture as compared with 0 and 6 days’ cultures, (b): Photomicrographs of chondrocyte-seeded constructs at different days of culture from the T-7 group; Note the increase in cell size with the progress of culture, Toluidine blue staining.

Histological evaluation of different chondrocyte-seeded constructs was carried out for 20 days of in-vitro culture. After 20 days, they were sectioned and stained for proteoglycan deposition using safranin-o. In each group, the culture positively stained for 20 days was compared with 0 and 6-day cultures and then the results were compared with those of 8 different constructs. The high positively stained proteoglycan deposition observed in the T-7 group is shown in Figure 6a. The morphological study showed that in 0-day cultures, chondrocytes appeared as ﬂattened cells without capsule and show a uniform distribution. A pericellular region of GAG-rich extracellular matrix, approximately 1 to 5 µm in width surrounded most cells on day 0. However, in 20-days culture, the cells had a rounded shape surrounded by a capsule and minimal cell division was observed. A glycosaminoglycan (GAG-rich) pericellular matrix, approximately 5 to 8 µm in width surrounded most cell groups. It appeared that the surrounding pericellular matrix in the T-7 group was obviously wider than the other groups (Figure 6a). The chondrocytes appeared to be increased in size by passing time and they had the largest size on day 20. Besides, the comparison between groups showed that the cell in the T-7 group had numerous cells with a larger size than the other groups (Figure 6b).

### Ultrastructural study

**Figure 7:**
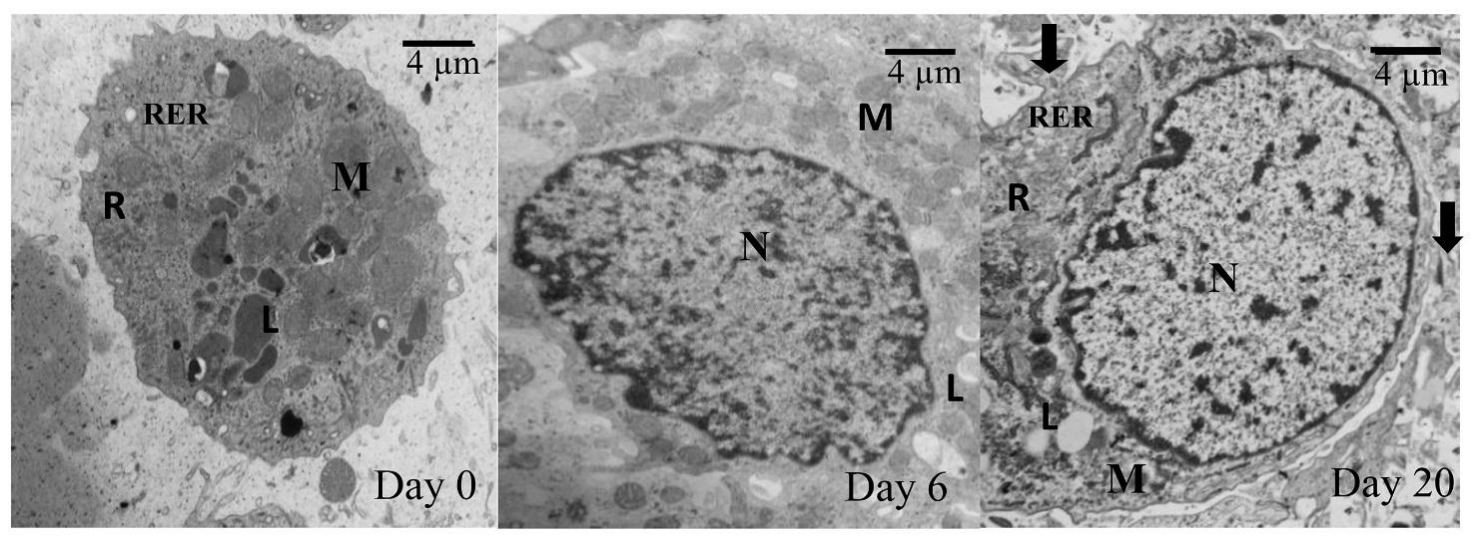
Transmission electron micrographs of chondrocytes at a different day of culture from the T-7 group; Note the appearance of collagen fiber (arrow) in the 20-days culture. Also, note chondrocyte characterized by Nucleus (N), Mitochondria (M), Rough endoplasmic reticulum (RER), Lipid droplet (L), Free ribosomes(R).

Transmission electron microscopy (TEM) revealed that the cells from different groups have similar ultrastructure. As shown in Figure 6a, in day 20, the cells within the constructs displayed all characteristics of chondrocyte (nucleus, lipid droplets, mitochondria, rough endoplasmic reticulum, and free ribosomes). Sparse and thin collagen type 2 fibers were found in ECM of 20-days culture. However, in 0-day culture (Figure 7), compared to 20-days culture, there were no collagen fibers in the ECM. Moreover, in 0-day, cells were morphologically flattened in the chondrocyte-seeded constructs.

### Cell Viability

**Figure 8:**
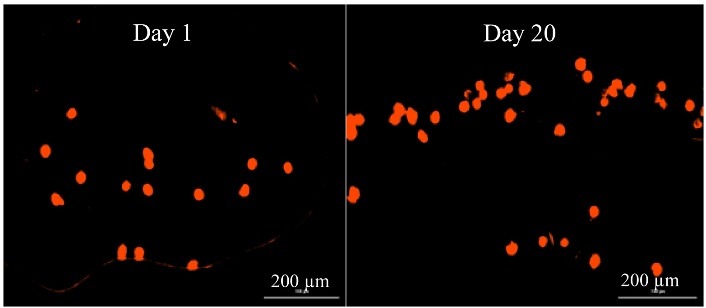
Cell viability of chondrocyte at different days of culture after printing by CM-Dil staining.

To determine cell viability after bio-ink extrusion, survival of chondrocytes was assessed using Dil dye. The chondrocyte viability and patterning were conducted by labeling with red fluorescent cell linker Dil dye. The viability of chondrocyte for a longer term in printed construct was assessed in 1-, 6-, and 20-days cultures (Figure 8). The printed cells continued to proliferate for 20 days after printing, with no significant difference when compared to the 1 and 6 days. Assessment of live cells by morphological studies showed ≥ 90% cell viability for 1-, 6-, and 20-days cultures after extrusion. Homogeneous red signals from the live/dead graph after 20 days of culture indicate that cells were populating the whole bioprinted construct. Figure 6b show viable cells on the bioprinted tracts immediately after printing. After 6 days of culture, the constructs were still filled with live cells (Figure 8). These results show that the printed construct preserved cell viability during the bioprinting process and provided a favorable microenvironment for cell proliferation. The highest live cells number were observed in the T-7 group (Table 4).

**Table 4:** Number of the viable cell after printing by day of culture in the T-7 group

Culture	Live cell number
1 day	108± 12.08
6 day	110.20±8.23
20 day	121.01±10.37

### WST assay and Cytotoxicity

**Figure 9:**
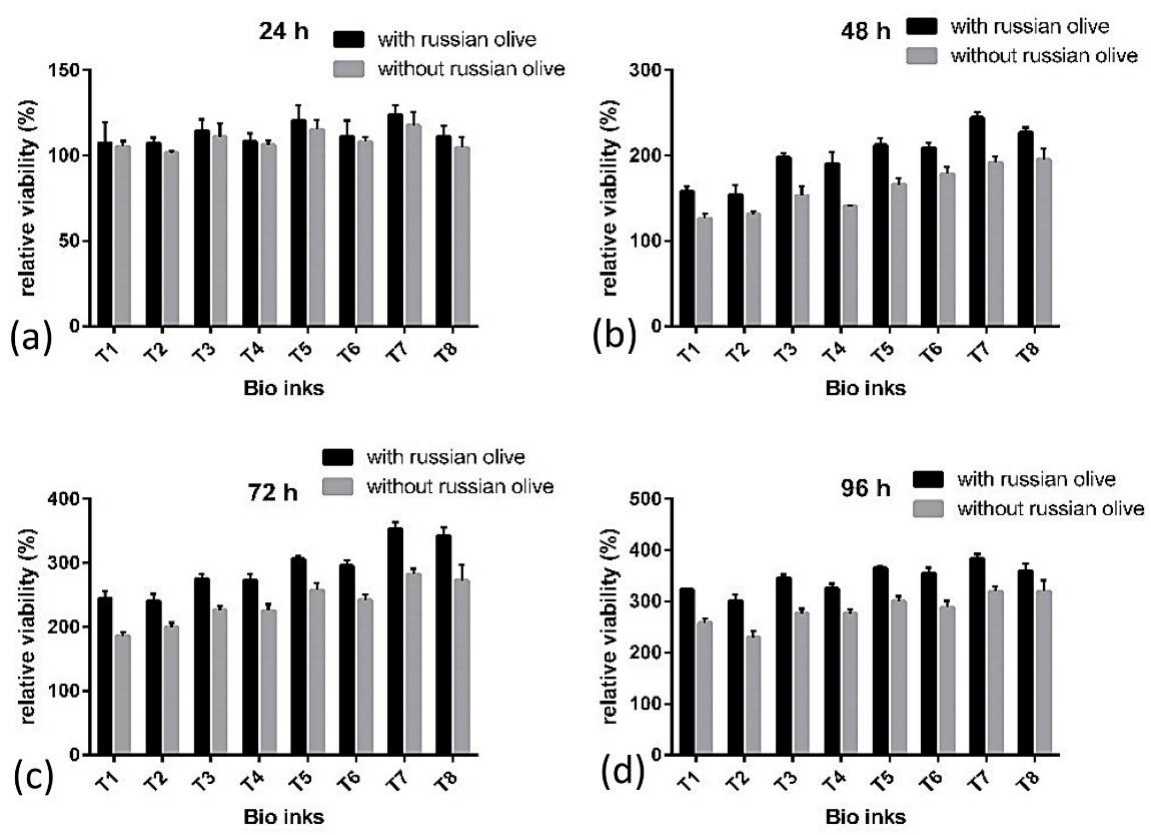
Relative viability percentage in a different composition cultured for a) 24 h, b) 48 h, c) 72 h, and d) 96 h.

Cell toxicity and proliferation in the bio-ink were tested by WST assay technique. A higher amount of alginate correlated with an increased cell viability percentage while a higher amount of HNT is associated with decreased cell viability percentage. The higher amount of alginate and lower amount of HNT in bio-ink correlated with better biological performance and showed a higher living percentage in WST assay tests. Presence of RO powder had a positive effect on cell viability percentage by 11%. To evaluate the effect of different composition on chondrocyte survival rate, the biomaterials were added to DMEM. Figure 9 shows the effects of different composition on viability that were cultured in DMEM for 24, 48, 72, and 96 h. All the experiments were performed in triplicate.

### In vivo study

**Figure 10:**
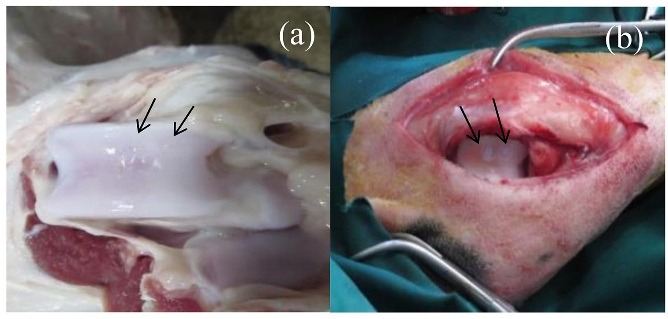
Post-surgical knee joint in implanted chondrocyte-seeded construct on cartilage defects after 6 months(a): The articular defects were repaired with cartilaginous tissues (b): Small amount of cartilage-like white color tissues at the defects in the control group after 6 months.

**Figure 11:**
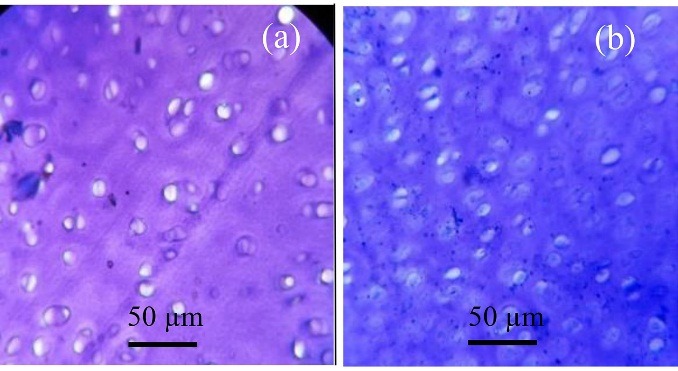
Photomicrograph from normal hyaline cartilage (a): Repaired tissue in implanted chondrocyte-seeded construct on cartilage defects after 6 months and (b): Toluidine blue staining.

**Figure 12:**
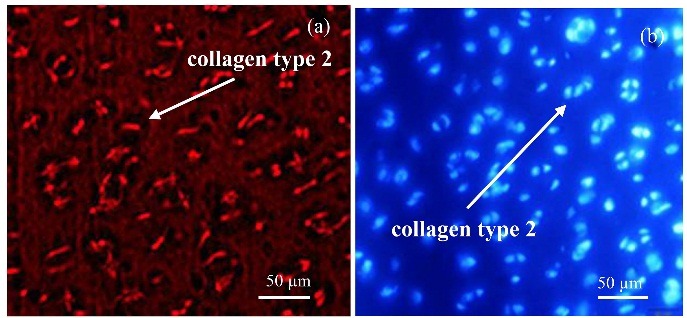
Immunofluorescence staining for expression of collagen type 2: (a): the chondrocyte-seeded constructs in articular defect after 6 months. (b): DAPI staining.

**Figure 13:**
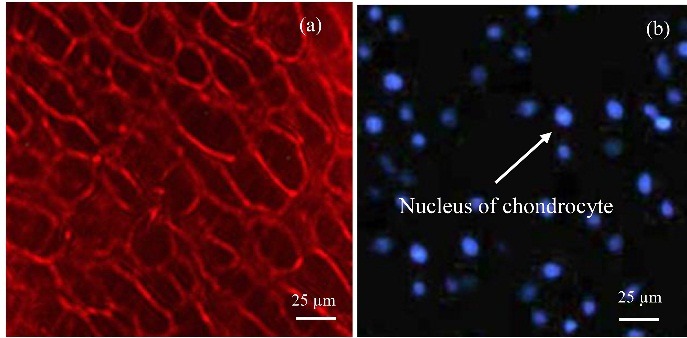
Immunofluorescence staining for expression of collagen Type 2 (a): In the negative control, (b): DAPI staining.

The in-vivo study was carried out based on in-vitro studies and the cells from the best responding group (T-7 group) were selected for the in-vivo study. Grossly, the repaired tissue in the artificial defect (Figure 10a) is hyaline cartilage-like when compared with normal hyaline cartilage (Figure 10b). The repaired cartilage tissue that resulted from a chondrocyte-seeded construct in treated sheep knees revealed hyaline cartilage-like tissue (Figure 11b) compared with normal hyaline cartilage (Figure 11a). Immunofluorescence staining exhibited a strong collagen type 2 positive staining and an increase in collagen type 2 in the chondrocyte-seeded construct (Figures 12a and 12b). Moreover, the negative control did not show any collagen type 2 expressions (Figures 13a and 13b).

Bio-inks with different compositions were printed, among which T-7 bio-ink showed the best results with chondrocytes. Different mechanical factors such as hardness, viscosity, and compressive properties influence the efficiency of bioprinted scaffolds. The proper hardness of bioprinted scaffolds should meet the hardness of natural cartilage. This range is from 0.5 to 4.0 MPa in microhardness tests.^6,27^ By increasing the amount of HNT in structure, shore A hardness of samples increases, probably due to the high mechanical characteristics of halloysite nanotubes and structure of halloysite.^28^ The high density of halloysite nanotubes and homogenous distribution of tubes along the applied force increase the hardness of biomaterials.^29^ The best results of hardness due to the mechanical and biological performance achieved in T-7 bio-ink. The bio-ink viscosity of biomaterials affects both printing and cell proliferation.^30,31^ From the printing aspect, the best kinematic viscosity for bioprinting of alginate-based bio-inks is between 2000 and 2400 mm^2^/s.^9^ A higher viscosity is required for a higher extrusion force, which in turn negatively affects living cells and causes problems such as printing needle blockage and lower resolution.^32^ In T-7 Bio-ink with proper viscosity, cell viability increases and leads to a higher bioprinting resolution. Alginate, HNT, and methylcellulose affect the bio-ink viscosity. Results of viscosity tests revealed that higher amounts of alginate and HNT increase the viscosity of bio-ink (Table 2). Alginate gel-like structure after crosslinking by CaCl_2_ and crosslink of halloysite nanotubes with each other and alginate are the reasons for a higher viscosity.^10^ Methylcellulose is added to bio-inks in cartilage scaffolds to adjust the viscosity of biomaterials.^12^ In higher viscosities, there are high pressures over cell-laden biomaterial for extruding from the needle; so the cells are harmed and cell viability is decreased. Results show that a proper viscosity that meets both biological and mechanical properties is T-7. In addition, when the viscosity is too low, the bioprinting resolution reduces significantly. By an increase in viscosity, printing resolution increased but the cell viability decreased. Hence, the viscosities from 2000 to 2400 mm^2^/s are the best choices in AL/MC/HNT/RO composition for using as a biomaterial in bioprinting process. The IR spectra of the bio-ink show the presence of all continents (AL, MC, HNT, and RO). The –OH peak at 3435.03 cm-^1^ is probably because of the presence of AL (20 mg/ml) in the composition.^33^ High concentration of HNT leads to a –C=O band at 1646.71, 1735.74, and 1561.93 cm-1.^33^ A peak at 2,930.00 cm^-1^ in the T-7 bio-ink is indexed to the aliphatic –CH groups existing in Alginate and MC. As mentioned earlier, MC was washed during the cell culture period and it produces a porous structure in scaffolds.^7^ These micro size porosities increase the cell living efficiency and improve cell attachments to bioscaffolds. Actually, the micropores produced along cell cultivation improves the surface potential of bioscaffolds for better cell adhesion.^34^ Compressive properties of bioscaffolds in T-7 Bio-ink meet the natural cartilage for achieving the best results.^35^ The human natural cartilage had a strength of 240-850 kPa in compression.^36,37^ Accordingly, bio-inks prepared in this study had a compressive stress between 241±45 kPa (T-1) to 839.66±10.01 kPa (T-8) and compressive stress for T-7 was 500.66±19.50 kPa. Compressive test results established that HNT had a remarkable role in increasing compressive properties and the bio-inks containing higher amounts of HNT had more desired compressive properties.^38^ As mentioned earlier, this phenomenon is due to the tubular structure of HNT and homogenous distribution of these tubules in all directions, which improve the strength of biomaterial. The effect of alginate is lower than that of the HNT on compressive properties because its gel-like structure after crosslinking and cross-linked Ca chains had lower compressive properties in comparison with HNT.^6^ WST assay tests results show high a percentage of living cells after 24, 48, 76, and 96 h according to compatible bio-inks composition with chondrocytes. Test results showed that cell viability increased from the first day of cell culture up to 96 h after bioprinting. It is evident from Figure 8 that relative viability is always higher in lower amounts of HNT and increases by increasing alginate in the composition. Additionally, the metabolic activity of chondrocytes is also increased in low HNT. Besides, as can be seen from GAG production, collagen type 2 is synthesized and grown in size by the passage of time. In the T-7 bio-ink that contains 30 mg/ml alginate and 30 mg/ml HNT, a higher percentage of cell viability (Figure 7) was achieved, suggesting the optimal property of prepared bio-ink for chondrocyte culture. Alginate is a well-known biomaterial used for bioscaffolding. On the other hand, HNT increases the printing and mechanical performance of bioprinted scaffolds.^30^ RO powder did not affect the mechanical properties of bioscaffolds but its fibrous structure changed the cell viability rate.^13^ Traditionally, RO fruit and seed powder are used to relieve pains in joints and knee. The sugar, vitamins such as tocopherol, vitamins C and B1, and minerals like calcium, magnesium, potassium, and iron existing in RO are essential for chondrocytes' growth.^15^ These properties of RO are the reason for a higher number of living cells when it is added to bio-ink. As can be seen from the WST results, RO affects the bioprinting cell viability by 11% after 96 h.

Another challenge with cartilage repair is that the proliferating chondrocytes tend to become fibrous cartilage, which is characteristically different from hyaline cartilage.^39^ Interestingly, in the present study, the cells maintained their hyaline cartilage property by producing collagen type 2 on 20 days of culture. From the mechanical and biological aspects, the T-7 bio-ink showed best results for bioprinting. The best bio-ink should meet both the mechanical and biological characteristics. Although mechanical properties of T-8 bio-ink was better than the T-7, higher amounts of HNT in its structure decreased the survival rate and bioactivity of seeded chondrocytes. T-7 bio-ink shows good mechanical properties and provides a proper medium for chondrocyte proliferation and bioactivity as shown by WST assay and morphological studies.

## Conclusion

Bioprinting of alginate/HNT/methylcellulose with RO powder was performed in this study. Mechanical and biological characterizations showed that composition containing 20 mg/ml alginate, 20 mg/ml methylcellulose, and 10 mg/ml halloysite nanotube solutions mixed with 10 mg/ml RO fruit and seed powder increased chondrocyte viability by 11%. Also, the compressive stress of bioprinted structures was increased by 210% in T-7 bio-ink in comparison with T-1 bio-ink, which contains lower amounts of alginate and HNT in its structure. Based on our results, it can be concluded that T-7 bio-ink has a high potential for usage in cartilage repairs and bioprinting process.

## Acknowledgments

This research did not receive any specific grant from funding agencies in the public, commercial, or not-for-profit sectors.

## Ethical Issues

All animals were treated in compliance with the “Principles of Laboratory Animals Care” formulated by the National Society for Medical Research. The study protocol was approved by the ethical committee of Tabriz University of Medical Sciences (No, IR. TBZMED.1395.1066).

## Conflict of Interest

The authors declare that they have no conflict of interest.
